# RETRACTED: Ahmed Althumairi et al. Recent Advances in Dielectric and Ferroelectric Behavior of Ceramic Nanocomposites: Structure Property Relationships and Processing Strategies. *Nanomaterials* 2025, *15*, 1329

**DOI:** 10.3390/nano16221453

**Published:** 2026-11-10

**Authors:** Nouf Ahmed Althumairi, Mokhtar Hjiri, Abdullah M. Aldukhayel, Anouar Jbeli, Kais Iben Nassar

**Affiliations:** 1 Department of Physics, College of Science, Majmaah University, Al-Majmaah 11952, Saudi Arabia; n.althumairi@mu.edu.sa (N.A.A.); a.aldukhayel@mu.edu.sa (A.M.A.); a.jbeli@mu.edu.sa (A.J.); 2 Department of Physics, College of Sciences, Imam Mohammad Ibn Saud Islamic University (IMSIU), Riyadh 11623, Saudi Arabia; mbhjiri@imamu.edu.sa; 3 Materials Physics Laboratory, Faculty of Sciences, Sfax University, BP 1171, Sfax 3000, Tunisia

The Journal retracts the review “Recent Advances in Dielectric and Ferroelectric Behavior of Ceramic Nanocomposites: Structure Property Relationships and Processing Strategies” [1] cited above.

Following publication, concerns were brought to the attention of the Editorial Office regarding the relevance and accuracy of several references cited in the study [1].

In accordance with standard journal procedures, the Editorial Office and Editorial Board conducted an investigation that identified a significant number of citations that were not sufficiently relevant to the article’s content and were not consistent with MDPI’s citation policies. Although the authors cooperated with the investigation, the explanations and additional materials provided were not deemed sufficient by the Editorial Board to resolve the identified concerns or support a correction. Consequently, the Editorial Board decided to retract this publication in accordance with MDPI’s retraction policy.

This retraction was approved by the Editor-in-Chief of the journal *Nanomaterials*.

The authors have been informed of this retraction and have indicated their disagreement with this decision.
